# Diet: Its Relation to Tooth-Tissue

**Published:** 1894-06

**Authors:** L. Ashley Faught


### DIET: ITS RELATION TO TOOTH-TISSUE.[1]

BY L. ASHLEY FAUGHT, D.D.S.

Mr. President and Gentlemen,—After the bountiful repast in which we have just indulged, how can I presume to say anything to you on the subject of diet. Your gustatory nerves, more than those of other members of the profession, are finely cultivated for the performance of their office. My permission lies in the knowledge that you always take with your dinners a little science as a digestive. The process of alimentation is chemical in its nature, and its relations to tooth-tissue have seemed to lie in an analytic study of foods and tooth-substance. Let us begin here. The carefully-prepared tables of the oft-quoted Bibra are before us. What do they teach? That enamel is largely inorganic, to the extent of ninety-six per cent.; that dentine is seventy-two per cent. inorganic and twenty per cent. organic. If we exclude for the moment the formative period of the teeth, a correct deduction is made that hopeless are all systemic dietary efforts to influence the condition of this outer tissue. Its destiny is largely a fixed quantity. With the dentine lie the greater possibilities. Our interest in it, therefore, cannot but be great. The maintenance of its normal calcific state is of utmost importance. As the inorganic percentage is lowered, its destruction by caries grows more imminent, and with the rise of organic percentage comes that great enemy "sensitive dentine," with its attendant neurotic evils. By the way, how foolish is this term we so frequently use! All healthy dentine is sensitive to a greater or less degree. Hypersensitive dentine more correctly expresses the condition. This is the fiend that unstrings the nervous system of the patient, exhausts the operator, limits too frequently the grand possibilities of dentistry, and has unjustly set pain and

---

[1] Read before the Central Dental Association of Northern New Jersey, December 18, 1893.

suffering in the public mind as the synonymes of our profession, though its proudest plume is the gift to humanity of anæsthesia.

Dentists have thus ever felt the apparent necessity of looking towards diet to obtain this needed supply of calcium phos. fluoride.

To continue our study, you and I must now make a chemical analysis of foods to ascertain those which are rich in that substance. But, as with tooth-tissue, so, too, has this been well done for us. We have but to refer to dietetic tables. What our patients shall eat and what our patients shall drink stands clearly presented. Our theoretic study is, therefore, complete. To patients with the indicative condition we prescribe this indicated food. How satisfactory should be the result!

But, gentlemen, it is not. Practice and theory do not so charmingly accord. Twenty years of careful study and observation have convinced me that such teaching is largely bosh and rubbish. Back of those twenty years (the progressive ones of dentistry) the fathers got into their heads the beautiful theory we together have just re-elaborated, and it has been handed down to the present moment as gospel truth. Go carefully through the literature and writings from 1872 to 1893, and be astonished at the beautiful pictures drawn in considering my topic by their ethnological studies. Time and time again is your attention directed to the beautiful teeth and to the diet of primitive peoples,—the Indian, negro, etc.,—and the deduction drawn,—like food, like teeth.

If you will but consider your experience in such practice, I am sure that your results have been even as mine,—largely a failure. You may stuff your patients full of such foods, and the result will be ever the same. The key to a change in the situation lies in a comprehension of assimilation. Only when the patients under this special care can be brought into a condition to assimilate the calcium phos. fluoride which you thus may present to their systems will you be able to upbuild tooth-tissue through such means, and only then to such degree as you may be able to restore the lost power of assimilation. The means at our disposal for the correction and control of assimilation are as many and as varied as the individual cases requiring attention, and in each instance must be governed by the case to be treated. It is impossible to expound any general procedure. It is to be borne in mind, however, that the modifications in the nutrition of the teeth due to food-supply have well-defined limits, and that their nutritive forces are to a large extent influenced by the condition of the nervous system.

A dental axiom in general terms and of wide application may thus be expressed. The prevalence of tranquillity of nerve establishes conditions promotive of assimilation, and a restless condition existing *in continuance* retards and perverts assimilation.

I have taken this occasion to present this one vital point of my topic, because, while it is appreciated as a general physiological truth, it has been in the past so sadly neglected, instead of being directly connected with all considerations of diet as affecting tooth-tissue.

If time permitted it would be a pleasure to me to now consider with you what I debarred at the outset,—diet during the developmental period of tooth-tissue, infant- and child-feeding; but I leave it to those who may take part in the discussion.

